# Clonal haematopoiesis of indeterminate potential is associated with progression of aortic valve stenosis

**DOI:** 10.1002/ejhf.70000

**Published:** 2025-08-06

**Authors:** Raúl Nicolas Jamin, Jasmin Shamekhi, Tobias Zeus, Victor Mauri, Muntadher Al Zaidi, Benedikt Bartsch, Ansgar Ackerschott, Georg Nickenig, Eicke Latz, Sebastian Zimmer, Baravan Al‐Kassou

**Affiliations:** ^1^ Heart Center Bonn, Clinic for Internal Medicine II University Hospital Bonn Germany; ^2^ Department of Cardiology, Pulmonology and Vascular Medicine University Hospital, Heinrich Heine University Düsseldorf Germany; ^3^ Heart Center, Department of Cardiology University Hospital Cologne Cologne Germany; ^4^ German Rheumatism Research Centre Berlin Germany; ^5^ Institute of Innate Immunity, University Hospital Bonn Germany

Aortic valve stenosis (AVS) is a prevalent and life‐threatening disease, which commonly initiates as clinically inapparent valve calcification or mild stenosis. Approximately 20% of these early patients develop severe AVS within 5 years, which is frequently associated with severe adverse events.^1^ Biomarkers that predict disease progression and allow risk stratification in early disease stages are currently lacking. Clonal haematopoiesis of indeterminate potential (CHIP) has been established as a risk factor associated with adverse outcomes in cardiovascular disease. Mechanisms in which CHIP‐positive leucocytes contribute to inflammation and fibrosis in cardiac disease have been demonstrated in extensive analyses, including single‐cell sequencing data sets.^1^, ^2^ Clinical data are however currently limited to patients with established severe AVS having received successful treatment via transcatheter or surgical aortic valve replacement, and have yielded inconsistent results on the association of CHIP with mortality.^3^, ^4^, ^5^ The influence of CHIP on AVS progression and related outcomes remains unclear.

This study aimed to evaluate the prevalence and prognostic relevance of CHIP in patients with early‐stage aortic valve disease.

Patients diagnosed with aortic valve sclerosis, mild or moderate AVS were recruited between 2018 and 2024 as part of the GUARD study at the University Hospital Bonn and followed up prospectively. Blood samples were obtained at the time of study inclusion. All patients provided written informed consent, the study was approved by the local ethics committee and was conducted in accordance with the Declaration of Helsinki. Clonal haematopoiesis (CH) was detected employing a targeted capture panel for 48 CH‐associated candidate driver genes using next‐generation sequencing on a NovaSeq 6000 (Illumina) with a sequencing depth of 20 000×, allowing detection of variants with an allele frequency of 0.5%. All patients harbouring a mutation in a candidate driver gene with a variant allele frequency (VAF) ≥2% (or 4% in X‐linked genes in men) remaining after pre‐processing and variant calling who did not fulfil criteria for myeloproliferative disease (World Health Organization definition) or active haematologic malignancy were considered CHIP‐positive. Progression was defined as an increase in AVS grading from baseline mild AVS or aortic valve sclerosis to at least moderate AVS, progression from baseline moderate to severe AVS, or progression to AVS requiring aortic valve replacement. The maximum clinical follow‐up period was set at 4 years, patients with no echocardiographic follow‐up were excluded from progression analyses (30 patients). AVS stages were defined according to the American College of Cardiology/American Heart Association guidelines.^6^ Statistical analysis was conducted using SPSS version 29.0.2.0 (IBM) and R Version 4.4.2. Univariate survival analyses were performed using a log‐rank Kaplan–Meier test, Cox regression analysis was conducted after verifying proportional hazard assumptions to account for possible confounding by baseline differences and clinical characteristics known to associate with AVS progression (age, sex, diabetes, hypercholesterolaemia, coronary artery disease, hypertension, chronic kidney disease). Significance was assumed if the *p*‐value (two‐sided) was <0.05, correction for multiple testing was conducted in exploratory analyses using false discovery rate.

We detected 597 CH driver mutations across 34 candidate driver genes in 224 out of 270 patients (83%), with 107 patients (39.6%) fulfilling the diagnostic criteria for CHIP. Consistent with existing literature, *DNMT3A* (109 patients [40.4%], VAF ≥2% in 38 patients [14.1%]) and *TET2* (81 patients [30%], VAF ≥2% in 18 patients [6.7%]) were the most commonly mutated driver genes, followed by mutations in *ASXL1*, DNA damage response (*TP53, PPM1D*) and spliceosome component (*SF3B1, SRSF2, ZRSR2, U2AF1*) genes (*Figure* *1A*). Patients with CHIP were numerically older (75.8 [95% CI 74.1–77.5] vs. 73.1 [95% CI 71.3–74.9] years, *p* = 0.101), more likely to be male (72 [67.3%] vs. 98 [59.9%] patients, *p* = 0.196) and to have a history of malignancy (31 [29%] vs. 32 [19.6%] patients, *p* = 0.076), although these differences did not reach statistical significance. Relevant comorbidities including hypertension, extent of coronary artery disease, valvular heart disease, chronic kidney disease or diabetes did not differ between groups. While high‐sensitivity C‐reactive protein was not significantly different between groups (2.57 mg/L vs. 2.27 mg/L, *p* = 0.232), patients with CHIP had a higher average leucocyte count (7.27 G/L vs. 6.78 G/L, *p* = 0.017). Parameters of AVS severity, including peak aortic velocity, peak gradient, mean gradient, and aortic valve area at baseline, did not differ significantly between groups. The study recruited 38 patients with aortic valve sclerosis, 153 patients with mild AVS and 79 patients with moderate AVS.

**Figure 1:**
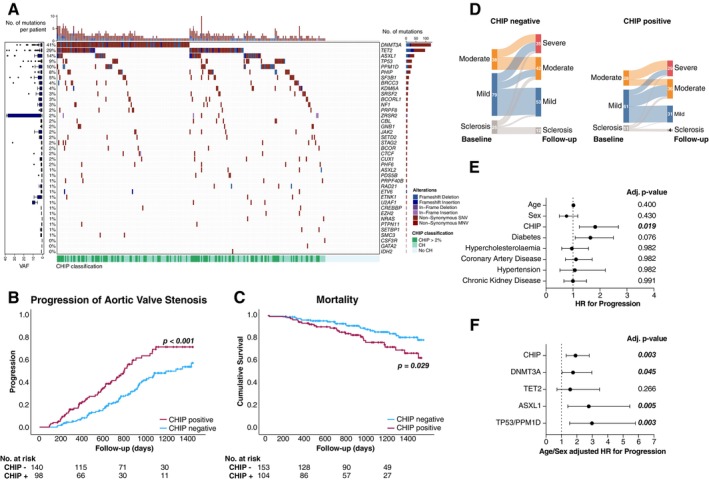
(*A*) Oncoplot of all mutations within the collective. (*B*) Univariate log‐rank Kaplan–Meier survival analysis for progression of aortic valve stenosis in clonal haematopoiesis of indeterminate potential (CHIP)‐negative versus CHIP‐positive patients. (*C*) Univariate log‐rank Kaplan–Meier survival analysis for mortality in CHIP‐negative versus CHIP‐positive patients. (*D*) Sankey plot of aortic valve stage progression in CHIP‐negative and CHIP‐positive patients. (*E*) Multivariable Cox regression analysis for progression of aortic valve stenosis for CHIP and other clinical factors. (*F*) Age/sex‐adjusted hazard ratio (HR) for progression for the most prevalent CHIP mutations. CH, clonal haematopoiesis.

Median echocardiographic follow‐up was 647 days (95% CI 546–732 days), median overall follow‐up was 894 days (95% CI 819–1002 days), follow‐up times did not differ between CHIP‐positive and negative patients.

During echocardiographic follow‐up, patients with CHIP were significantly more likely to suffer a progression of AVS (54 patients with CHIP [55.1%] vs. 54 patients without CHIP [38.7%], *p* < 0.001 [univariate log‐rank Kaplan–Meier]) (*Figure* *1B*). Median progression‐free survival time was 806 days (95% CI 870–1103 days) in patients with compared to 1301 days (95% CI 969–1623 days) in patients without CHIP. Patients with CHIP also displayed significantly increased mortality in a VAF‐dependent manner, with 26 patients with CHIP (24%) having died during follow‐up, compared to only 22 (13.7%) of patients without CHIP (*p* = 0.029, univariate log‐rank Kaplan–Meier) (*Figure* *1C*). While patients with CHIP were more likely to die due to malignancy (5 [20.8%] vs. 2 [9.1%] deaths), cardiovascular mortality was similar between groups (6 [25%] deaths in CHIP‐positive and 7 [31.8%] deaths in CHIP‐negative patients).

Within the follow‐up period, 29 (30%) CHIP‐positive patients progressed to severe AVS or AVS requiring intervention/surgery compared to 36 (24.5%) patients without CHIP. Additionally, 26 (26.3%) CHIP‐positive patients progressed to moderate AVS, compared to 19 (13.5%) CHIP‐negative patients (*Figure* *1C*). Dividing the binary progression outcome by the follow‐up time in years to calculate an annualized progression rate per patient for the overall collective yielded a mean progression index of 0.29 for patients without and 0.56 for patients with CHIP (*p* < 0.001). After adjusting for differences in age and sex, as well as taking into account other clinical influences potentially associated with AVS progression using multivariable Cox regression analysis, the association of CHIP and progression of AVS remained statistically significant (adj. *p* = 0.019; hazard ratio [HR] 1.822, 95% CI 1.236–2.685) (*Figure* *1E*). The HR for patients with CHIP to suffer a progression was higher than for all other factors examined, and CHIP was the only factor to be significantly associated with progression risk after correction for multiple testing. When including all patients with CH driver mutations at VAF <2%, CH showed no significant association with AVS progression (*p* = 0.224), and VAF of the largest clone did not correlate with progression risk (*p* = 0.558). Multivariable Cox regression analyses revealed diverging age/sex‐adjusted HRs to suffer AVS progression for different mutations (*Figure* *1F*). While progression rates in patients with CHIP due to *DNMT3A* (HR 1.754, 95% CI 1.037–2.969, adj. *p* = 0.045), *ASLX1* (HR 2.771, 95% CI 1.414–5.432, adj. *p* = 0.005) or p53 pathway mutations (*TP53, PPM1D*) (HR 2.979, 95% CI 1.531–5.798, adj. *p* = 0.0025) were significantly increased compared to patients without CHIP, *TET2* CHIP was not significantly associated with AVS progression (HR 1.569, 95% CI 0.709–3.472, adj. *p* = 0.266). CHIP‐positive patients with three or more mutations showed a numerical increase in progression rates compared to CHIP‐positive patients with one–two mutations, which did not reach statistical significance (39.3% vs. 51.2% progression free survival, *p* = 0.371).

Our results show that CHIP is not only highly frequent in AVS but serves as a predictive marker for disease progression. We observed that CHIP is a highly prevalent phenomenon in patients with lower‐grade AVS, occurring in 39.6% of cases – markedly higher than the rates expected for their corresponding age group. CHIP, but not CH, was significantly and robustly associated with the risk to suffer a progression of AVS, leading to higher rates of advanced disease requiring valve replacement in CHIP‐positive patients. In contrast, other clinical characteristics thought to be implicated in AVS progression were not significantly associated with progression. Multivariable analysis revealed marked differences in progression risk between mutations, with *DNMT3A*, *ASXL1* and *TP53/PPM1D* mutations showing individually significant associations with AVS progression, with a tendency for higher progression rates in patients with multiple mutations. CHIP was further associated with mortality in a VAF‐dependent manner. Overall mortality in the collective was relatively low and information on cause of death not available for all patients, therefore statistical assumptions regarding differences in causes of death between patients with and without CHIP are difficult to draw. Long‐term compounding effects of CHIP on AVS specifically and cardiovascular disease‐related outcomes generally might manifest prospectively and aggravate differences.

These findings provide important evidence for the clinical relevance of CHIP as a biomarker to predict AVS progression and give clinical relevance to mechanistic connections discovered between CHIP and molecular and cellular pathways of AVS progression. Due to the limited sample size and consequently limited number of patients with specific driver mutations as well as possible confounding due to overlapping effects between multiple mutations within one patient, results will have to be replicated in larger multicentre clinical cohorts to more accurately assess the effects of specific driver mutations on disease progression.

## Funding

This work was funded by the Deutsche Forschungsgemeinschaft (DFG, German Research Foundation) (grant no. 397484323 ‐ TRR259).

**Conflict of interest**: S.Z. has received speaker fees from AstraZeneca, Medtronic, Abiomed, Edwards, Boehringer Ingelheim, Daiichi Sankyo, Boston Scientific, Novartis, Abbott, Pfizer, ACIST, Bristol Myers Squibb, Bayer, Boston Scientific. All other authors have nothing to disclose.
